# Regenerative Immunotherapy for Cancer: Transcription Factor Reprogramming of Tumor-Specific T Cells

**DOI:** 10.3390/cancers17132225

**Published:** 2025-07-02

**Authors:** Tyler R. McCaw, Nicholas P. Restifo, Kathrin Plath, Joseph G. Crompton

**Affiliations:** 1 Division of Surgical Oncology, University of California Los Angeles, Los Angeles, CA 90095, USA; tmccaw@mednet.ucla.edu; 2 Broad Stem Cell Research Center at UCLA, University of California Los Angeles, Los Angeles, CA 90095, USA; kplath@mednet.ucla.edu; 3 Medici Therapeutics, Boston, MA 02114, USA; 4 Department of Biological Chemistry, University of California Los Angeles, Los Angeles, CA 90095, USA

**Keywords:** transcription factor reprogramming, T cell exhaustion, stemness, immunotherapy, adoptive cellular therapy

## Abstract

Cell-based immunotherapy is a promising treatment strategy for cancer. Particularly in the case of solid tumors, however, this strategy only benefits a minority of patients. A critical limitation to immunotherapy is T cell exhaustion, a terminal differentiation state characterized by loss of self-renewal and cytotoxic capacity. For over a decade, regenerative immunology approaches to overcome exhaustion and restore stem-like features of T cells have been pursued. The reprogramming of tumor-specific T cells back to a less-differentiated, stem-like state using induced pluripotent stem cell (iPSC) technology has been viewed as a powerful and highly appealing strategy to overcome the limitations imposed by exhaustion. However, clinical translation of these approaches has been stymied by the requirement for subsequent iPSC-to-T cell re-maturation strategies, vanishingly low efficiencies, and resource-intensive cell culture protocols. In this review, we discuss the emergence of transcription factor reprogramming to iPSCs, contemporary techniques for T cell reprogramming, as well as techniques for re-differentiation into mature T cells. We discuss the potential clinical utility of T cell reprogramming and re-maturation strategies alongside progress and major roadblocks toward clinical translation. If these challenges can be addressed, transcription factor reprogramming of T cells into iPSCs and subsequent re-maturation into tumor-specific stem-like T cells may represent an incredibly efficacious approach to cancer immunotherapy.

## Simple Summary

Immunotherapy has revolutionized cancer treatment. However, these strategies are contingent upon the quality of a patient’s pre-existing anti-tumor immune response, limiting the clinical efficacy. T cell exhaustion—a cellular program characterized by loss of stemness and anti-tumor effector functions—critically limits the quality of a patient’s tumor-specific T cell repertoire and thereby their likelihood of experiencing clinical benefit from immunotherapy. Transcription factor reprogramming of tumor-specific T cells (with subsequent maturation techniques) is envisioned as a strategy to restore stemness and functionality to a patient’s tumor-specific T cell repertoire and turn an immunotherapy non-responder into a responder. Unfortunately, current T cell reprogramming and re-maturation protocols require extended culture periods and are prohibitively resource-intensive. Herein, we discuss the state of these approaches alongside progress toward clinical application.

## 1. Introduction

Cancer is a complex amalgam of (pre-)malignant cells, recruited cells, and environmental factors. In some ways, the progression of a cancer reflects Darwinian evolution, wherein cellular clones possessing enhanced fitness are preferentially selected. However, this view largely negates the contribution of environmental factors, particularly tumor cell niches, epigenetic reprogramming, and cellular plasticity, recent additions to the Hallmarks of Cancer [1]. Indeed, environment-imposed plasticity—put forth in extended evolutionary synthesis—is now recognized as a significant contributor to malignant progression [2]. It follows that effective treatment strategies must be equally sophisticated and ideally able to undergo parallel evolution with the cancer. Current cancer treatment strategies often incorporate different modalities, traditionally including chemotherapy, radiation, and surgery. Although the goal of oncologic surgery is to remove 100% of the tumor(s), this is often not possible due to pre-existing occult metastases and/or spatial relatedness of the tumor(s) to critical anatomic structures. Chemotherapy and radiation, non-operative modalities, were initially pursued for their ability to somewhat selectively damage rapidly dividing cancer cells [3]; the efficacy of this approach is constantly advancing through targeting a broader range of critical mechanisms and drug delivery strategies [4]. Importantly, the utility of these agents is increasingly ascribed to their (re-)stimulation of anti-tumor immune responses and restructuring of the tumor microenvironment [5,6,7]. Patient-derived xenograft models—transplanting patient tumor tissue into severely immunocompromised mice—were initially developed as an in vivo strategy to study potential drug efficacies and mechanisms; however, a lack of a native immune system (including secondary immune organs) significantly curtails the utility of these models for the abovementioned reasons [8]. Strategies focused specifically on engaging anti-tumor immunity, or immunotherapy, have now revolutionized cancer treatment. Earning the moniker of a “living drug”, adaptive immune cell-based therapies are able to engraft in a patient, persist for many years, and instigate an immune response able to undergo parallel evolution with the tumor (particularly through clonal spreading [9]). Thus, immunotherapeutic strategies and their incorporation into rational combinatorial strategies afford a promising and necessary degree of sophistication in the pursuit of curative cancer therapies.

T cell-based immunotherapy is an auspicious treatment strategy for patients with solid tumors, but currently benefits only 10–20% of patients [10,11]. In the case of adoptive cellular therapy (ACT), autologous circulating CD8 T cells can be isolated and engineered with a chimeric antigen receptor or T cell receptor, allowing them to target a single tumor antigen. Alternatively, a primary or metastatic tumor can be surgically resected, and tumor-specific polyclonal CD8 T cells isolated, expanded, and reinfused into the patient. An additional benefit of isolating T cells from the tumor is that these are polyclonal, targeting a host of tumor antigens. These antigens can be private (unique to the patient) or public (frequently shared by patients with a particular cancer histology); however, it is not necessary to know antigen identity, as T cells isolated from tumors are (assumed to be) largely tumor-specific. Complete and durable responses are possible with ACT; however, these are infrequent and, when they do occur, require engraftment of rare stem-like T cells that can persist and self-renew [12,13]. A critical limitation to this approach is that most of a patient’s tumor-specific T cells that can be harvested from a resected tumor are terminally differentiated or exhausted. This greatly limits their ability to expand and generate the billions of cells needed for ACT. While CD8 T cells available in the blood are typically not exhausted, they are very rarely tumor-specific. Therefore, there is a clinical need to develop strategies that promote tumor-specific T cell stemness and overcome exhaustion.

Transcription factor-based cellular reprogramming is a powerful technique that may be equal to these ambitious goals, generating stem-like, tumor-specific T cells for clinical application. In this review, we will discuss the emergence of transcription factor reprogramming, the evolution of T cell reprogramming, in vitro strategies for T cell maturation, as well as the current status of clinical translation.

## 2. T Cell Differentiation and Loss of Stemness

T cell precursors egress from the bone marrow to the thymus, wherein they progress through well-defined developmental stages. Initially, a double negative T cell is formed (CD4^−^ CD8^−^, DN), wherein the T cell receptor (TCR) β chain rearranges, then forms a double positive cell (CD4^+^ CD8^+^, DP) with corresponding TCR α chain rearrangement, then a mature single positive (CD4^+^ or CD8^+^) naïve αβ T cell. Upon initial antigen encounter in the periphery (e.g., tumor-draining lymph node in the context of cancer), naïve T cells become activated and begin to differentiate. In a linear model of T cell differentiation, T cells first differentiate into stem central memory, followed by central memory, effector memory, and effector [14]. Repeated antigen encounter, suppressive signaling, and a myriad of environmental factors in the tumor microenvironment (TME) can then drive T cell terminal differentiation into the exhaustion program. Moreover, T cells can potentially exit this typical linear differentiation model and progress toward an exhausted fate at any point given sufficient stimuli [15]. The concept of immune exhaustion can be traced to experiments in the 1960s and 1970s using high doses of antigen exposure and describing depletion or exhaustion of a memory immune response [16,17]. Ultimately, the mechanistic underpinnings of this observation were elucidated with the discovery of inhibitory immunologic checkpoint surface proteins [18,19]. Demonstration of differential transcriptional regulatory circuits between memory versus exhausted CD8 T cells revealed a central role for the transcription factor TOX in the establishment of exhaustion [20]. Persistent TCR engagement and calcineurin signaling—encountered during chronic infections and cancer—leads to prolonged activation of NFAT family proteins, culminating in expression of TOX, detectable within 24 h of antigen exposure. The expression of TOX correlates with that of the suppressive proteins PD1, TIGIT, LAG3, CTLA4, TIM3, CD160; the transcription factors TCF1 and Eomes; and the loss of effector cytokines. Importantly, the TOX program becomes at least partially independent of NFAT signaling once established. One reason for this may be that TOX binds to, or recruits, epigenetic modifiers including the HBO1 complex, DNMT1, LEO1, PAF1, SAP130, and SIN3A to participate in chromatin restructuring [21]. These findings have spurred attempts to curtail TOX expression in favor of preserved functional capacity. TOX loss prior to priming may initially preserve effector functions in the context of chronic viral infection [22], but the same was not observed in a murine-inducible liver cancer [23]. Nevertheless, inducible TOX loss after establishment of the exhaustion program only permits partial fate flexibility, with recovery of some effector gene expression toward an effector-like state [24]. This may be due to the cooperative action of TOX, NFAT, and NR4A transcription factors in driving the exhaustion fate [25].

We now understand T cell exhaustion to be an epigenetically mediated differentiation step that constrains stemness, proliferation, and effector functions [26]. Moreover, tumor-specific T cells adopt this program extremely rapidly, beginning within 6 h of initial activation [27]. Even after removal of cognate antigen, e.g., with complete surgical resection of a cancer, elimination of HCV facilitated by direct-acting antivirals, or ex vivo culture, exhausted CD8 T cells are unable to re-acquire the stem-like capacity of their less differentiated counterparts [28]. So, while less differentiated stem-like T cells are required for successful ACT, most of a patient’s tumor-specific T cell repertoire is either exhausted or has already initiated that program.

**Figure 1:**
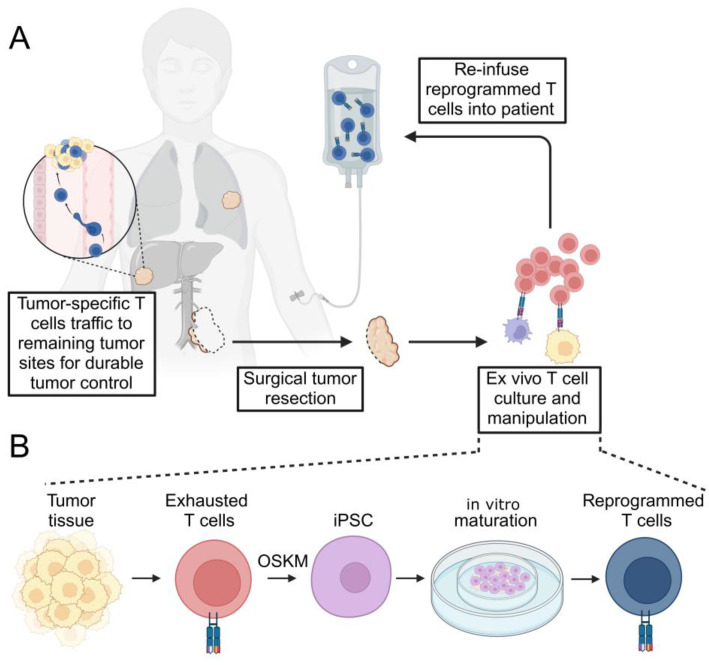
Schematic of tumor-specific T cell rejuvenation for clinical application. (**A**) Depicted here is the resection of a primary tumor (which may be incomplete due to proximity to critical anatomic structures). Infiltrating, tumor-specific T cells are selectively cultured ex vivo with the opportunity for therapeutic manipulation. The expanded T cell product is then re-infused into the patient in an autologous fashion. Reprogrammed T cells traffic to sites of remaining tumor and secondary lymphatic tissues (not depicted) to exert durable tumor control. (**B**) Terminally differentiated, exhausted tumor-specific T cells isolated from surgically resected tumor specimens are subjected to transcription factor reprogramming to generate T-iPSCs. Pluripotent cells are then re-differentiated into mature, reprogrammed T cells in vitro using a variety of described methods (depicted here using an artificial thymic organoid, ATO). OSKM—Oct4, Sox2, Klf4, c-Myc.

Because exhaustion is an epigenetically mediated phenomenon, efforts have intensified over the past decade to use stem cell technology to overcome exhaustion. Here, terminally differentiated somatic cells of any lineage can be de-differentiated to fully recover stemness and pluripotency through the introduction of specific transcription factors. In this process, the epigenome of a differentiated cell is reset to an embryonic-like state, allowing re-expression of stem and progenitor genes. Importantly, prior genomic rearrangements of the T cell receptor (and B cell receptor) are preserved. The result is that the epigenome can be reset, while the T cell receptor specificity is maintained so that the suppressive epigenome enforcing T cell exhaustion can be erased and stemness recovered. Here, T cells are reprogrammed to induced pluripotent stem cells (iPSCs) and then re-differentiated into mature T cells (Figure 1). Although conceptually a powerful and highly appealing approach, it has been practically challenging to accomplish.

## 3. Early Reprogramming Strategies

As an organism ages, tissue-specific stem cells, their niches, and appropriate regulation are progressively lost. Consequently, tissue function is impaired, marked by an eroded capacity for self-repair and homeostasis. Although these processes affect all tissues, we will focus herein specifically on hematologic lineages.

Cellular reprogramming represents an attractive strategy to recover stemness. Early attempts at reprogramming took the form of nuclear transfer and cellular fusion techniques—approaches intended to test whether genomic information is conserved during development. Seminal experiments demonstrated that nuclei transferred from adult keratinocytes into enucleated eggs of the same species could support amphibian development to the tadpole stage, but no development to an adult was observed [29]. Although this demonstrated formation of all three germ layers and subsequent tissue maturation, it highlighted the question of whether permanent genetic changes may be incurred during development that ultimately curtail a cell’s ability to be reprogrammed into a truly pluripotent (or totipotent) cell. Nuclear transfer of a mammary gland cell from an adult ewe into an enucleated oocyte reconstructed embryos that, when implanted into a recipient ewe, facilitated development to term and live birth of a lamb with characteristic morphological features, thereby demonstrating that genetic material for full reprogramming to pluri- and totipotency are retained during normal development [30]. The ability of nuclei from terminally differentiated somatic cells to be reprogrammed to an embryonic state and support mammalian development to term was then redemonstrated in mice [31].

Cell–cell fusion of undifferentiated human embryonic stem cells (hESCs) and fibroblasts or hESC-derived myeloid precursors demonstrated the ability of a hESC to reprogram the transcriptional state of somatic nuclei to an embryonic state. Although this generates a tetraploid cell, hybrid cells were able to re-differentiate into myeloid precursors with similar efficiencies as diploid hESCs [32,33].

Lymphocytes—both T and B cells—were initially reprogrammed by nuclear transfer to test the hypothesis that nuclei of terminally differentiated cells could be reset to pluripotency and that prior successes at animal cloning with nuclear transfer were not owed to the transfer of rare somatic stem cells present in heterogeneous donor populations. Mature T and B cells afforded a convenient tool in this regard, as rearranged TCR and BCR loci imbrued a genetic barcode of terminal differentiation that could be traced before and after reprogramming. Although less efficient than reprogramming with other terminally differentiated lineages, both T and B cells were able to be reprogrammed through a modified two-step approach to generate embryonic stem cell lines [34]. This proof of principle for lymphocyte reprogramming by nuclear transfer was then extended to include natural killer T cells [35]. Collectively, these experiments begged the question of whether cell-intrinsic reprogramming could be achieved solely through the identification and ectopic expression of defined genes and factors [36].

## 4. Contemporary Techniques for T Cell Reprogramming

A paradigm shift occurred in 2006, when cellular reprogramming was achieved using forced expression of four transcription factors—Oct4, Sox2, c-Myc, and Klf4 (OSKM)—in mouse [37] and human [38] fibroblasts. Starting with a list of 24 candidate genes involved in the establishment and/or maintenance of pluripotency, a systematic combination of factors revealed that fibroblasts can be reprogrammed through ectopic expression of Oct4, Sox2, c-Myc, and Klf4. Separately, starting with a list of 14 genes involved in the establishment and/or maintenance of pluripotency, lentiviral transduction of subsets of these genes revealed fibroblast reprogramming could also be achieved with OCT4, SOX2, NANOG, and LIN28 (though sequencing of generated iPSC clones demonstrated LIN28 to be dispensable) [39]. Importantly, transcription factor reprogramming drives global reversion of the somatic epigenome to an embryonic state. Bisulfite sequencing demonstrated DNA methylation of the Oct4 and Nanog promoters to be similar to that of reference embryonic stem cells (ESCs), while chromatin immunoprecipitation of H3K4 and H3K27 trimethylation revealed these histone modifications in iPSCs to be highly concordant with ESCs. Functionally, iPSCs derived from murine embryonic fibroblasts could give rise to chimeric offspring when injected into diploid blastocysts [40].

Shortly thereafter, transcription factor-based reprogramming was redemonstrated by others in human [41] and mouse cells [42]. A consistent and emerging observation was that more differentiated cells exhibited lower reprogramming efficiency than less differentiated cells, requiring additional measures to achieve reprogramming to iPSC [39]. For example, increased efficiency of more differentiated cells was achieved with co-transduction of hTERT and SV40 (though the mechanism of this was postulated to be indirectly on reprogramming cells) [41]. These cells adopted a characteristic human ESC-like morphology, expression of stem cell markers by IHC and PCR (including SSEA3, SSEA4, Tra-1-60, Tra-1-81, Nanog, hTERT, REX1), and formation of teratomas comprised of all three germ layers upon injection into severely immunocompromised mice.

Importantly, several groups demonstrated the applicability of this technology for reprogramming mature T cells. Loh et al. used a lentiviral approach, transducing twice eight days apart, to reprogram peripheral blood mononuclear cells (PBMCs) with an efficiency of 0.0008–0.001%. The generated iPSC colonies expressed OCT4, NANOG, Tra-1-60, Tra-1-81, and SSEA4 by IHC as well as hTERT, REX1, and GDF3 by PCR. Pluripotency was demonstrated by teratoma formation with all three germ layers. Given the heterogeneity of PBMCs, PCR then demonstrated TCR-delta rearrangement to confirm that at least some of the iPSCs generated originated from a T cell lineage [43]. Transduction with retroviral OSKM similarly generated iPSC from mature mouse T cells when p53 was genetically ablated (but no colonies were observed in wild-type p53), occurring at an efficiency of 0.00055% [44]. The reprogramming efficiency of T cells to iPSC was improved when retroviral-based transduction was replaced with Sendai-virus transduction. Sendai virus is a negative-sense RNA virus that does not integrate and remains in the cytoplasm and yields strong, transient expression of the reprogramming factors [45]. Sendai-based reprogramming using only OSKM achieved an efficiency of 0.1% when starting with PBMCs cultured under T cell-enriching conditions [46].

## 5. The Vision of T Cell to iPSC Reprogramming

The ability to reset the epigenome of a terminally differentiated T cell to an ESC-like state using iPSC technology was then envisioned as a platform with which to revitalize anti-tumor immunity and make immunotherapy a more efficacious strategy for patients with cancer [47]. That is, because T cell exhaustion is an epigenetically enforced differentiation program, reverting tumor-specific exhausted T cells into iPSCs will erase the suppressive epigenome of exhaustion while preserving TCR specificity. Although the T cell reprogramming attempts discussed to this point used circulating T cells, the frequency of tumor-specific T cells possible to be isolated from blood is vanishingly low (unless subjected to further selection protocols).

An alternative strategy is to reprogram T cells that have infiltrated the tumor mass, as these are more likely to be tumor-specific by virtue of having been recruited to and retained in the tumor. In this way, polyclonal tumor-specific T cells could be expanded, or further selection of T cells specific for known tumor antigens can be pursued. As a first step toward clinical application, iPSC technology was used to reprogram tumor-infiltrating lymphocytes (TILs) isolated from patients with melanoma. Vizcardo et al. reprogrammed a TIL cell line derived from a patient with melanoma, JKF6 cells, which possess specificity for MART-1, a public melanoma antigen. Transduction with Sendai-encoding OSKM plus SV40 yielded an efficiency of 0.004%. The generated iPSCs expressed SSEA4, Tra-1-60, and Tra-1-81 by immunofluorescence, and could form teratomas with all three germ layers. TCRVβ gene rearrangements confirmed that the iPSCs were, in fact, derived from mature T cells with previously rearranged TCRs [48]. Next, Saito et al. reprogrammed primary TILs, which are more differentiated than their circulating counterparts (which the authors confirmed by flow cytometric analysis of CD45RO, CCR7, PD1, TIM3, and Lag3). Transduction with Sendai-OSKM generated iPSCs expressing canonical markers SSEA3, SSEA4, OCT4, Tra-1-60, and Tra-1-81 and capable of forming all three germ layers. The generated iPSCs were also found to harbor a variety of TCRβ chain rearrangements, reflecting a polyclonal culture. Notably, however, TIL cultures were not sorted for CD3 or CD4/8, nor for more or less differentiated subsets present in culture prior to infection [49]. Therefore, less differentiated cells (or residual non-T lineages) in culture may be disproportionately represented in generated iPSCs. Reprogramming efficiency when using TILs, however, tends to be lower than historical reports reprogramming lymphocytes isolated from blood. To address this limitation, the reprogramming efficiency of tumor-specific TILs isolated from cultured tumor fragments (wherein TILs egress from tumor tissue over time in culture) [50] was shown to be enhanced by TCR stimulation prior to transduction with Sendai-OSKM and concurrent Sendai-SV40 [51].

**Figure 2:**
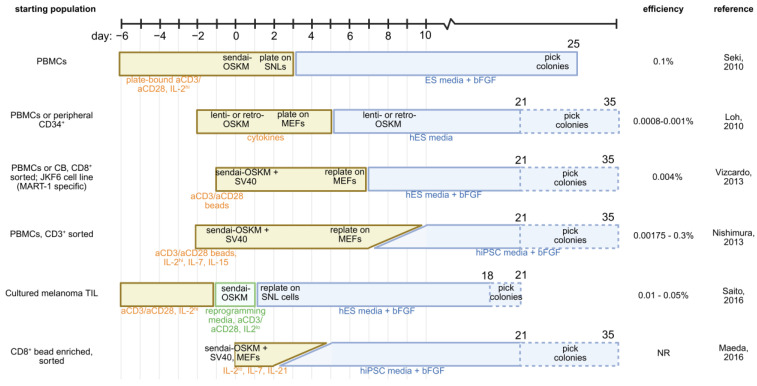
Comparison of described strategies for transcription factor-mediated reprogramming of T cells into iPSCs. Starting cell population and reported reprogramming efficiencies are provided. Yellow boxes represent culture in T cell media; blue boxes represent transition to stem cell media; green boxes represent reprogramming-specific media [43,46,48,49,56,57]. PBMC—peripheral blood mononuclear cell; IL-2—interleukin 2; IL-7—interleukin 7; IL-15—interleukin 15; ES—embryonic stem cell; bFGF—basic fibroblast growth factor; OSKM—Oct4, Sox2, Klf4, c-Myc; MEF—mouse embryonic fibroblast; SV40—large T antigen; SNL—SNL76/7 mouse embryonic fibroblast cells.

These studies collectively demonstrate that reprogramming tumor-infiltrating T cells, which are more differentiated than circulating T cells, requires additional efforts to increase efficiencies (e.g., inclusion of SV40). This is in line with historical reports that more differentiated cells of the hematopoietic lineage reprogram less efficiently [52]. Technological advances in OSKM delivery continue to improve reprogramming efficiency [53], which is crucial when the translational goal is to preserve the tumor-specific T cell repertoire. To this end, it may be that other, yet unexplored, transcription factor combinations are better suited specifically for T cell reprogramming [54]. Additional methods for cellular reprogramming and rejuvenation continue to be advanced, including cytokines and growth factors, autophagy modulation, epigenetic manipulation with small molecules, metabolic modulation, and synthetic biology approaches. These techniques have recently been comprehensively reviewed [55]. A summary of currently reported protocols for reprogramming of mature T cells to iPSC is provided in Figure 2.

## 6. T Cell Reprogramming Necessitates Subsequent Differentiation and Maturation Techniques

De-differentiation of tumor-specific T cells using nuclear transfer or transcription factor-mediated reprogramming is able to overcome the suppressive epigenome, enforcing T cell exhaustion. However, the result of T cell reprogramming is a T cell-derived iPSC. Although these iPSCs (theoretically) maintain their original TCR rearrangements, in the iPSC state, they lack T cell functions, including tumor-directed trafficking and antigen-specific cytotoxicity. Therefore, T cell-derived iPSCs must be re-differentiated into mature T cells for the regeneration of a clinically useful therapeutic.

Early attempts at redifferentiation of T cell-derived iPSCs used a murine bone marrow stromal cell line, OP9. Co-culture of hESCs on OP9 cells could give rise to hematopoietic progenitor cells with the potential to differentiate into the T cell lineage when injected into thymic or fetal liver implants in SCID mice [58]. Upon trafficking to the thymus, T cell progenitors receive notch signaling, which is critical for thymic T cell maturation [59,60]. In vitro differentiation of hematopoietic progenitors into single- and double-positive TCRαβ and γδ T cells was then achieved via ectopic expression of delta-like ligand 1 (DLL1) on OP9 cells (OP9-DLL1) [61]. This strategy was subsequently adapted to the differentiation of human ESCs into mature TCRαβ T cells [62]. When using cells without a previously rearranged TCR, this co-culture method induces RAG-mediated rearrangement to produce a broad TCR repertoire [63]. When starting with CD8 T cell-derived iPSCs (CD8-iPSCs) with previously rearranged TCRs, co-culture with OP9-DLL1 cells regenerates largely antigen-specific CD8 T cells (although loss of antigen specificity does occur, as discussed below) with cytotoxic capabilities. For example, using virus-specific CD8 T cells from an HIV-1-infected patient, Sendai-OSKM plus Sendai-SV40 generated CD8-iPSCs, which could be re-differentiated into mature CD8 TCRαβ T cells using OP9-DLL1 cells. The resultant “rejuvenated” T cells did not express PD-1 but sometimes (co-)expressed CCR7, CD27, and CD28, suggesting a naïve or central memory phenotype. The “Rejuvenated” cells were significantly more proliferative than the T cells of origin (20- versus 100–1000-fold expansion in culture) and produced canonical cytokines (IFNγ and granzyme B) upon antigen stimulation [56]. Similarly, iPSCs derived from a long-term melanoma TIL culture could be re-differentiated on OP9-DLL1 cells to generate functional CD8 T cells, many of which retained the original antigen specificity [48]. Additional attempts to optimize the OP9-DLL1 system of T cell maturation later followed. Ascorbic acid increases the differentiation of thymic progenitors into DP and SP T cells [64], and is now commonly included in T cell differentiation media. The efficiency of the DP to CD8 SP transition can be increased by adding soluble anti-CD3 antibody, culturing maturing CD8 T cells on RetroNectin, and supplementing IL-7, IL-15, and IL-21. Because CD8αβ T cells generated using the OP9-DLL1 monolayer still retain innate-like features, sorting on NKp44-negative and CD8αβ-positive and CD5-positive cells improved the yield of CD8αβ T cells in culture [65]. For ease of culture, OP9-DLL4 cells have also been co-transduced to express typical components of T cell differentiation media: IL-7, FLT3L, and SCF [66].

However, the OP9-DLL1/4 system is subject to several critical limitations. First, T cell-derived iPSCs (T-iPSCs) are able to undergo an additional round of recombination. Vizcardo et al. noted that approximately 30% of maturing T cells weakly or do not recognize cognate antigen at the DP stage of re-maturation (although the culture was enriched to about 95% antigen-specific again by the SP CD8 T cell stage) [48]. Iriguchi et al. found a similar loss of antigen specificity at the CD8αβ SP T cell stage following differentiation of T-iPSC specific for several antigens [67]. Minagawa et al. further demonstrated that TCR transduced iPSCs differentiating on the OP9-DLL1 system began re-expressing RAG1/2 mRNA by the second week, with peak expression by week 3–4. This correlated with additional TCRα rearrangements at the DP stage and loss of antigen specificity in 40% of matured CD8αβ T cells. However, this additional TCR recombination could be prevented by CRISPR-mediated knockout of RAG2 [68]. Second, the OP9-DLL1 culture system is inefficient and requires approximately 4–6 weeks or more for maturation to CD8 SP T cells [62,67], followed by additional time for the expansion of the generated T cells. Most importantly, OP9-DLL1 stromal cell-mediated differentiation primarily generates innate-like CD8αα T cells. These CD8αα cells have weaker cytotoxicity than conventional CD8αβ, as determined by peptide concentrations required in cell killing assays [57,68], consistent with historical data of impaired TCR signaling [69]. Additionally, CD8αα cells generally fail positive selection [70] and have limited persistence in vivo [71]. The generation of CD8αα T cells was ascribed to the direct cytotoxicity of DN cells (which give rise to CD8αα) against DP cells (which have the potential to generate CD8αβ T cells) during differentiation [57]. The selection of DP cells (i.e., removal of DNs from culture) mitigated this effect to generate CD8αβ T cells, though these cells still retained some innate-like features, had an effector-like profile, and exerted less cytotoxic capacity than canonical CD8αβ T cells. The characterization of CD8 T cells produced by the OP9-DLL1 system using RNA-sequencing suggested these cells represent an immature CD8 SP stage with high expression of NK cell markers, low MHCI, and persistent RAG expression [71].

Transition from two-dimensional to three-dimensional (3D) culture systems marked a major advance in the regeneration of naive-like CD8αβ T cells. Conceptually, 3D culture more closely recapitulates the architectural environment sensed by a maturing T cell as it migrates through a native thymus. First, a 3D fetal thymic organoid culture system was constructed by harvesting a murine fetal thymus at day 15.5 of gestation, lymphodepleting with 2-deoxyguanosine, and placing thymic tissue into a suspended media droplet. Thymic lobes were then seeded with iPSCs cultured on OP9-DLL1 cells for 16–21 days, which were predominantly DP and DN cells at that point. This protocol generated so-called “iPSC-derived thymic emigrants” (iTEs). When compared to OP9-DLL1 culture-derived CD8 T cells, iTEs demonstrated superior antigen-specific proliferation and cytokine production. RNA-sequencing demonstrated iTEs to be more similar to naïve CD8 T cells than OP9-DLL1 derivatives. Functionally, upon adoptive transfer of small numbers of Pmel-specific iTEs (10^4^ cells), these cells were able to form central memory subsets (though to a lesser extent than naïve T cells), expand in vivo, and control tumor growth similar to (albeit statistically significantly less than) naïve T cells [71]. The use of primary thymic tissue, however, presents logistical challenges, variable outcomes, and is not feasible for clinical applications.

Therefore, the development of an artificial thymic organoid (ATO) subsequently proved another significant advance in T cell maturation techniques, affording a fully defined and reproducible culture system. Here, the mouse bone marrow stromal cell line MS5 expressing human DLL1 (MS5-DLL1) is admixed with human progenitor cells with T lineage potential—including HSPCs, bone marrow lymphoid progenitors, primed multipotent progenitors, and CD24^−^ common lymphoid progenitors—pelleted (conferring 3D structure), and the culture is supported by micropore filters at the air liquid interface. Each ATO generates approximately 2 × 10^6^ total cells at different stages of maturation by week 6, and, compared to the OP9-DLL1 monolayer system, this technique generates significantly more DP and CD8αβ TCRαβ T cells at 4 and 6 weeks. T cells produced in ATO culture have a naïve phenotype (CD45RA^+^ CD45RO^−^, CD27^+^, CCR7^+^, CD1a^lo^, CD62L^+^, CD28^+^) and, upon activation, express CD25 and 4-1BB, proliferate, and produce IL-2, TNFα, and IFNγ. Importantly, both the 3D structure and media used dictated efficient, positive selection of DP precursors to generate conventional CD3^+^ CD8αβ TCRαβ T cells [72]. The human ATO system also supports T lineage commitment and mature CD8αβ TCRαβ T cell production when starting with iPSCs [73]. The authors then extended their work by developing a murine ATO, enabling more mechanistic pre-clinical studies. Here, they used the MS5 stromal cell line now expressing DLL4 (MS5-DLL4) and similarly pelleting with hematopoietic stem progenitor cells (Lin^−^, Sca1^+^, cKit^+^, or other hematopoietic progenitors) and culturing on a membrane at the air–liquid interface. Similarly, each ATO generated approximately 2–3 million total cells at different stages of maturation by 3 weeks. The generated CD8 SP and CD4 SP cells displayed a broad repertoire, as well as proliferated and produced canonical T cell cytokines in response to stimulation [74].

**Figure 3:**
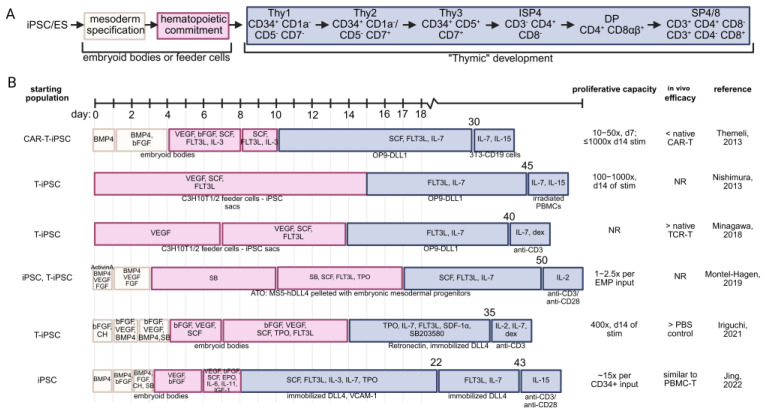
Comparison of the described in vitro T cell maturation strategies without the use of primary thymic tissues. (**A**) T lineage commitment is first accomplished by inducing iPSC or embryonic stem cells toward mesoderm commitment, followed by hematopoietic commitment. This is typically performed by culturing on feeder cells or using low-attachment dishes to generate embryoid bodies. To mimic native thymic signaling, CD34^+^ hematopoietic cells receive notch signaling through a variety of strategies. Depicted are the canonical stages of T cell development, which are recapitulated to differing degrees by the listed strategies. (**B**) Schematic representation of strategies for differentiation of iPSC into mature T cells. Starting cell population, estimated proliferation, and relative in vivo efficacy (as reported) of the regenerated T cell product are provided [56,67,68,73,76,77]. BMP4—bone morphogenetic protein 4; bFGF—basic fibroblast growth factor; SCF—stem cell factor, FLT3L—FMS-like tyrosine kinase 3 ligand; IL-3—interleukin 3; IL-7 interleukin 7; IL-15—interleukin 15; DLL1/4—delta-like ligand 1/4; CH—CHIR99021, a GSK-3 inhibitor; SB—SB431542, a TGF-βRI inhibitor; dex—dexamethasone; NR—not reported; TPO—thrombopoietin; SDF-1α—stromal cell derived factor 1 (CXCL12); EPO—erythropoietin; VCAM-1—vascular cell adhesion molecule 1; EMP—embryonic mesodermal progenitor.

Given regulatory hurdles and challenges with clinical translation, more recent efforts to increase high efficiency and fidelity maturation of CD8αβ T cells have increasingly focused on the development of serum- and feeder-free systems. Intending to generate a more reproducible approach, such a platform was generated by culturing the CD34^int^ CD43^+^ fraction of differentiating human embryoid bodies on immobilized DLL4 with RetroNectin in the presence of CXCL12 and a p38 inhibitor (SB203580) to generate CD8αβ T cells. The majority of these cells retained original antigen specificity [67]. Alternatively, human CD34^+^ hemogenic endothelial cells isolated from embryoid bodies can be cultured on DLL4- and VCAM-1-coated dishes in the presence of SCF, FLT3, IL3, IL7, and TPO, giving rise to a population of CD5^+^ CD7^+^ T cell progenitors. Continued culture on DLL4-coated plates with FLT3 and IL7 generated DP cells that can be differentiated into SP T cells via anti-CD3/anti-C28 TCR stimulation and IL-15. shRNA-mediated knockdown of EZH1 during T cell specification then significantly increased the formation of CD8αβ T cells, with minimal emergence of CD8αα T cells, resulting in increased CD8 SP after 6 weeks of stroma-free culture conditions. RNA sequencing demonstrated serum- and feeder-free T cells (with or without EZH1 knockdown) to be more similar to peripheral blood TCRαβ T cells than their OP9-DLL1-derived counterparts, downregulating innate-like gene expression (e.g., TRDC, KLRB1). Single-cell RNA-sequencing demonstrated maturation of naïve-like (CCR7^+^ SELL^+^ IL2RA^lo^ LEF1^hi^), effector-like (CCR7^−^, SELL^−^, GZMB^hi^, NKG7^+^), and memory-like T cells (CCR7^+^, SELL^+^, IL7R^+^, CD2^hi^, CCL5^hi^, FAS^hi^, EOMES^hi^) within the mature T cell compartment [75]. Starting with human iPSCs, the same group demonstrated inhibition of the histone lysine methyltransferase G9a to promote lymphoid and T cell fate at the expense of myeloid development [76]. A summary of reported protocols for re-differentiating iPSCs into mature T cells is provided in Figure 3.

## 7. Current Models and Applications for T Cell Reprogramming

### 7.1. Personalized Versus “Off the Shelf”

The characteristic features of iPSCs—including self-renewal, pluripotency with its broad differentiation potential, and capacity for unlimited expansion—seemingly invite the application of the iPSC technology to adoptive cellular therapy of cancer. Because billions of cells must be generated for adoptive cellular therapy, “off-the-shelf” approaches confer the benefits of reduced costs and time required with standardized therapeutic product quality. However, some important considerations remain. For these iPSC-derived T cells to be clinically useful, they must recognize ubiquitous or public cancer antigens, either through use of lineages with restricted repertoires or transgenically directed T cell specificity (e.g., chimeric antigen receptor (CAR) or TCR). Although the immense potential of using defined antigens has been demonstrated in metastatic colorectal cancer [78] and metastatic pancreatic cancer [79], the requisite knowledge of tumor antigens and the putatively limited prevalence of public or “shared” tumor antigens in a patient population presents a significant obstacle [80,81]. In an off-the-shelf approach, transferred T cells conceivably would still need to be HLA-matched for at least one allele, necessitating iPSC stocks ideally homozygous for each HLA haplotype [82]. An alternative approach to avoid immune rejection of reprogrammed cells and/or graft-versus-host disease is the concurrent administration of immunosuppressive drugs, thereby increasing associated clinical toxicities.

### 7.2. Use of Immune Lineages with Restricted Antigen Recognition

Applicability of transcription factor reprogramming was extended to demonstrate that CD4^+^ and CD4/CD8 double-negative iNKT cells could be reprogrammed to iPSCs using Sendai-OSKM and Sendai-SV40 [83]. Similarly, Vγ9Vδ2 T cells were successfully reprogrammed to iPSCs [84]. The reprogramming of T cell lineages that make use of restricted TCR rearrangements is thought to provide a potential avenue for an “off-the-shelf” approach, wherein iPSC-derived from (for example) iNKT or γδ T cells can kill target cells through either innate or adaptive ligand recognition (i.e., in the context of HLA molecules).

### 7.3. Directing Antigen Specificity Using TCR Transgenes

One approach to broaden the utility of an iPSC starting cell population is to transduce iPSCs with the TCRα and TCRβ chains specific for a known, public tumor antigen. This was demonstrated to be feasible when using iPSCs derived from T cells or non-T cells, followed by lenti-TCR transduction at the pluripotent stage. Interestingly, re-maturation on OP9-DLL1 cells resulted in significantly greater efficiency of CD8αβ T cell generation when starting with T cell-derived iPSCs [85]. This may reflect epigenetic memory of iPSC for their cell of origin [86], or perhaps an incomplete reprogramming of so-called iPSC to an ESC-like state. Moreover, iPSCs derived from different CD8 T cell clones recognizing a single antigen (e.g., MART-1) demonstrated significant variability in potential to re-differentiate into DP and CD8αβ T cells [87]. Nonetheless, non-T cell-derived iPSCs can be transduced with a TCR of known specificity to generate HLA-restricted, antigen-specific CD8αβ T cells with in vitro cytotoxicity [88] and in vivo efficacy (e.g., renal cell carcinoma PDX model) [89]. The efficacy of this approach is potentially limited by expression of endogenous TCR chains (with unintended positive selection through mispairing or intact endogenous TCR rearrangements) as well as additional RAG1/2-mediated recombination. It follows that high-fidelity selection of antigen-specific CD8αβ T cells matured from human pluripotent stem cells can be achieved through CISPR-mediated knockout of RAG1, RAG2, and B2M; transduction of an exogenous TCR (e.g., targeting NYESO); and expression of the cognate human MHC in co-cultured stromal cells. Indeed, such CD8 SP T cells expressing a single, exogenous TCR demonstrated superior tumor control relative to matured CD8 T cells with intact endogenous loci [90].

### 7.4. Directing Antigen Specificity Using Chimeric Antigen Receptors

Chimeric antigen receptors fuse the antibody variable fragment with intracellular T cell signaling domains, including at least CD3ζ, and later generations including co-stimulatory (CD28 or 4-1BB) and other functional domains [91]. The combination of iPSC and CAR technology, then, is envisioned to harness the proliferative potential of iPSC technology as well as the ability to engineer target cell specificity in an HLA-unrestricted fashion with CAR technology. In this way, a cell line of CAR-engineered iPSC-derived T cells could be maintained with the ability to treat patients irrespective of HLA restriction. The feasibility of this approach was first demonstrated by Themeli et al. [77]. The authors used retroviral-OSKM transduction of peripheral T cells, then transduced T-iPSC with a lentiviral CD19-specific second-generation CAR with CD28 and CD3ζ signaling domains. CAR T-iPSCs were then re-differentiated on OP9-DLL1 cells. However, matured T cells were more innate-like, resembling γδ T cells, expressing CD8α but not CD8β. Nonetheless, re-differentiated T cells were able to elaborate IL-2, TNFα, and IFNγ upon CD19^+^ target cell encounter and control lymphoma growth in SCID mice on par with CAR transduced TCRαβ or TCRγδ cells.

## 8. Techniques for CD4 T Cell Reprogramming and Re-Maturation

CD4 T cells are increasingly recognized to provide critical help to CD8 T cells in ACT approaches. Indeed, when CD4 and CD8 T cells are co-transduced in CAR T cell preparations, the combined product has increased clinical efficacy [92,93]. This may be due to increased persistence of CAR T cells as well as direct cytotoxic capabilities of CD4^+^ CAR T cells [94]. It follows that the generation of iPSC-derived T cell therapies will likely also benefit from the co-transfer of regenerated CD4 and CD8 T cells. The ATO system can generate mature TCRαβ CD4 SP cells, though the frequencies of these are far less than CD8 SP development [72]. Thus, there is an unmet need for strategies to repurpose in vitro differentiation methods toward CD4 SP T cells. Conveniently, the activation with phorbol 12-myristate 13-acetate (PMA) and ionomycin can provide moderate and transient increases in intracellular Ca^2+^ and PKC activity, inducing commitment of DP thymocytes to the CD4 lineage without TCR engagement [95]. Fong et al. activated iPSC-derived DP T cells with PMA and ionomycin to generate CD4 SP T cells, expressing CD45RA^−^, CD4RO^+^, CD28^+^, and CD62L^lo^, suggestive of an effector memory phenotype. Notably, by single-cell RNA-sequencing, iPSC-derived CD4 SPs were distinct from their primary T cell counterparts (with iPSC-derived CD4 SPs expressing a profile similar to type 3 innate lymphoid cells) and failed to produce IFNγ on stimulation [96]. To better understand barriers to CD4 fate commitment, a time course of single-cell RNA-sequencing comparing iPSC-to-T cell maturation via DLL4-coated plates (2D), the ATO system (3D), and primary thymocytes was conducted. Pseudotime analysis implicated insufficient activity of the transcription factor ZBTB7B (or Thpok, known to antagonize the CD8 program and permit CD4 T cell development [97]) as well as gene candidates LZTFL1 and SOX4 [98]. Further work is needed to identify additional strategies and their integration for more efficient iPSC-to-CD4 T cell maturation. In this way, iPSC-derived adoptive cellular therapies could similarly benefit from the inclusion of tumor-specific CD4 and CD8 T cells.

## 9. Progress as a Clinical Therapy and Major Roadblocks to Translation

Since the first report of human iPSC derivation in 2007, there have been several approaches to clinical translation focusing on retinal pigment epithelial cells for macular degeneration; insulin-producing beta cells for diabetes; cardiomyocytes for heart failure; and neurons for spinal cord injury (for a recent review, see reference [99]). The use of regenerative immunology approaches to improve the efficacy of T cell immunotherapies for cancer is broadly relevant for the treatment of hematologic and solid malignancies as well. CAR therapy is currently used to treat lymphoma and multiple myeloma, TIL therapy to treat melanoma, and transgenic TCR therapy to treat synovial cell sarcoma.

Using the iPSC technology to improve current T cell-based immunotherapies (e.g., ACT, TIL, CAR, and transgenic TCR therapies) has at least a couple of advantages. The first way regenerative immunology can potentially improve T cell therapy for cancer is by rejuvenating exhausted anti-tumor T cells through transcription factor reprogramming and subsequent re-maturation (as discussed previously). Second, all FDA-approved cell-based immunotherapies are autologous products (at the time of this publication). Autologous therapies are exorbitantly expensive, resource-intensive, and suffer from variability in cell production. Therefore, allogeneic cell therapy products for cancer are appealing because of the potential for an “off-the-shelf” product that is relatively cheaper, more scalable, and has consistent quality in the cell manufacturing. Thus, an “off-the-shelf” application of iPSC-to-T cell maturation targeting public antigens may be particularly advantageous.

There are at least two early-phase clinical trials using iPSC-derived T cells in an “off-the-shelf” capacity, both sponsored by Fate Therapeutics. A phase I study (NCT04629729) is evaluating an allogeneic iPSC-derived CAR T cell product targeting CD19 in patients with relapsed or refractory B-cell lymphoma. The CAR construct is inserted in the T cell receptor (TCR) alpha constant (TRAC) locus to eliminate the endogenous TCR and potentially decrease the risk of graft-versus-host disease, while rendering T cells CD19-specific. Although this trial has not been completed, there are reportedly complete remissions in some patients and a favorable safety profile after adoptive transfer of 90–360 million cells (https://ir.fatetherapeutics.com/news-releases/news-release-details/fate-therapeutics-announces-clinical-safety-and-activity-data, accessed on 23 March 2025). Another clinical trial (NCT06241456) is evaluating an “off-the-shelf” allogeneic iPSC-derived T cell product targeting human epidermal growth factor receptor 2 (HER2) in patients with advanced solid tumors that over-express HER2. This therapeutic product has seven genetic modifications, including a HER2 CAR integrated into the endogenous TRAC locus and a synthetic IL-7/IL-7 receptor fusion to promote T cell stemness [100]. Clinical performance and overall patient outcomes have not been subject to peer review and will come into focus as the data matures.

To date, there are no allogenic iPSC-derived products with an endogenous TCR or a genetically modified TCR that are being evaluated in clinical trials. The main obstacle in translating iPSC-derived T cell products with an endogenous TCR or genetically modified TCR to the clinical setting is creating scalable cell manufacturing that can reliably provide an environment of thymic development. Moreover, and of particular concern in “off-the-shelf” approaches, transgenic TCRs may mispair with endogenous chains, creating a possibility of alloreactivity, graft-versus-host disease, and loss of tumor specificity [101]. As mentioned previously, reactivation of RAG1/2 can also compromise original TCR specificity. Perhaps for these reasons, proportionately more iPSC-derived NK cellular products are currently being pursued in (primarily phase I) clinical trials, some of which are also transduced with CAR constructs to confer antigen-specificity. Applications of iPSC and ESC-derived cellular products for a range of pathologies have recently been comprehensively reviewed [102].

Despite the potential advantages, a key concern remains that generating iPSCs from TILs and then re-differentiating them into T cells is extremely slow, resource-intensive, and logistically daunting. One reason is that this requires a clinical facility capable of producing GMP-grade iPSCs for a single patient (in the case of autologous approaches). With current de-differentiation and re-differentiation approaches, it would take several months (4–6 months) for manufacturing, too long for patients with advanced cancer who need an urgent intervention. Moreover, as diseases progress for patients during the manufacturing process, the heterogeneity of tumor metastases might render their autologous product ineffective because it no longer contains a repertoire that is capable of targeting new metastases. Although TIL-derived iPSCs have been generated at a small scale, translating this approach to manufacturing billions of clinical-grade T cells for each patient is an unsolved challenge. Another real-world problem is that generating iPSCs under GMP conditions and then re-differentiating them with 3D thymic organoids or feeder-free protocols is massively resource-intensive. The cost and complexity barriers remain so high that it could be a long time before we see iPSC-derived TIL products on the market unless solutions to cost, speed, and safety can be developed. Moreover, several key regulatory hurdles have yet to be addressed in the production of these therapies at a clinically relevant scale, including protecting against off-target actions, preventing insertional mutagenesis, ensuring cellular product purity, and possible teratoma formation if less differentiated cells persist in the final product [103].

## 10. Future Directions

The regeneration of a functional tumor-specific T cell repertoire via transcription factor reprogramming to iPSCs and subsequent re-maturation has transformative potential for patients with solid tumors and chronic infections. Importantly, because T cell exhaustion currently delimits the efficacy of immunotherapy for many patients, reprogramming approaches may greatly expand the number of patients clinically benefitting from immunotherapy. However, in addition to the hurdles discussed above, several considerations must yet be addressed prior to broader clinical implementation.

Clinical safety of the cellular product upon patient administration is paramount, and this remains a critical concern for regenerative techniques to date. Transition to Sendai-based transgene delivery alleviates concern regarding deleterious insertion sites. As an alternative approach, the development of RNA-based strategies for iPSC induction, including a commercially available non-integrating synthetic polycistronic RNA replicon [104], is anticipated to improve the safety of reprogrammed cellular products [105]. Interestingly, this approach may actually have repercussions on immune surveillance, as modification of RNA bases might lead to subsequent malignant progression [106], frameshifting [107], or promote antigenicity and interferon signaling [108,109]. With final product safety in mind, increasing incorporation of chemical reprogramming has been pursued as a strategy to improve safety and precisely tailor cell fate decisions [110]. Beyond insertional mutagenesis, incomplete differentiation protocols and residual undifferentiated iPSCs similarly pose a clinical safety concern. Here, persistence of Sendai-OSKM in a final cell product conceivably preserves multipotency and/or fate plasticity, carrying a risk of malignant transformation in vivo. This can be addressed by co-transfer of a “suicide gene”, or inducible caspase-9, into reprogrammed iPSCs, which facilitates rapid killing of the final cellular product at a specific timepoint [111]. Although each of these strategies addresses particular safety concerns of cellular reprogramming, they can be laborious in nature and significantly compromise the efficiency (or yield) of the final cellular product.

## 11. Conclusions

Great promise has been demonstrated by applying transcription factor reprogramming with subsequent re-maturation to T cell-based immunotherapy strategies. Indeed, the ability to regenerate tumor-specific T cells with naïve- and memory-like phenotypes represents a monumental advance in immunotherapy. Moreover, the vast replicative capacity of iPSCs facilitates the production of clinically relevant cell numbers. Nonetheless, since the conception of this approach, excitement has been tempered by currently untenable costs, the requirement for robust infrastructures, and extended culture periods. If the abovementioned obstacles to clinical translation can be effectively addressed, then the addition of reprogramming strategies to the oncologic armamentarium may be embraced with renewed enthusiasm.

## References

[1] Hanahan D. (2022). Hallmarks of Cancer: New Dimensions. Cancer Discov..

[2] Savy T., Flanders L., Karpanasamy T., Sun M., Gerlinger M. (2025). Cancer evolution: From Darwin to the Extended Evolutionary Synthesis. Trends Cancer.

[3] DeVita V.T., Chu E. (2008). A history of cancer chemotherapy. Cancer Res..

[4] Liu B., Zhou H., Tan L., Siu K.T.H., Guan X.Y. (2024). Exploring treatment options in cancer: Tumor treatment strategies. Signal Transduct. Target. Ther..

[5] Galluzzi L., Buqué A., Kepp O., Zitvogel L., Kroemer G. (2015). Immunological Effects of Conventional Chemotherapy and Targeted Anticancer Agents. Cancer Cell.

[6] Rückert M., Flohr A.S., Hecht M., Gaipl U.S. (2021). Radiotherapy and the immune system: More than just immune suppression. Stem Cells.

[7] Charpentier M., Spada S., Van Nest S.J., Demaria S. (2022). Radiation therapy-induced remodeling of the tumor immune microenvironment. Semin. Cancer Biol..

[8] Gu A., Li J., Li M.Y., Liu Y. (2025). Patient-derived xenograft model in cancer: Establishment and applications. MedComm (2020).

[9] Aoki H., Tsunoda M., Ogiwara H., Shimizu H., Abe H., Ogawa T., Abe T., Shichino S., Matsushima K., Ueha S. (2023). Clonal Spreading of Tumor-Infiltrating T Cells Underlies the Robust Antitumor Immune Responses. Cancer Immunol. Res..

[10] Mao Y., Xie H., Lv M., Yang Q., Shuang Z., Gao F., Li S., Zhu L., Wang W. (2023). The landscape of objective response rate of anti-PD-1/L1 monotherapy across 31 types of cancer: A system review and novel biomarker investigating. Cancer Immunol. Immunother..

[11] Osipov A., Lim S.J., Popovic A., Azad N.S., Laheru D.A., Zheng L., Jaffee E.M., Wang H., Yarchoan M. (2020). Tumor Mutational Burden, Toxicity, and Response of Immune Checkpoint Inhibitors Targeting PD(L)1, CTLA-4, and Combination: A Meta-regression Analysis. Clin. Cancer Res..

[12] Gattinoni L., Lugli E., Ji Y., Pos Z., Paulos C.M., Quigley M.F., Almeida J.R., Gostick E., Yu Z., Carpenito C. (2011). A human memory T cell subset with stem cell–like properties. Nat. Med..

[13] Biasco L., Izotova N., Rivat C., Ghorashian S., Richardson R., Guvenel A., Hough R., Wynn R., Popova B., Lopes A. (2021). Clonal expansion of T memory stem cells determines early anti-leukemic responses and long-term CAR T cell persistence in patients. Nat. Cancer.

[14] Gattinoni L., Klebanoff C.A., Restifo N.P. (2012). Paths to stemness: Building the ultimate antitumour T cell. Nat. Rev. Cancer.

[15] Henning A.N., Roychoudhuri R., Restifo N.P. (2018). Epigenetic control of CD8^+^ T cell differentiation. Nat. Rev. Immunol..

[16] Byers V.S., Sercarz E.E. (1968). The X-Y-Z scheme of immunocyte maturation. IV. The exhaustion of memory cells. J. Exp. Med..

[17] Sercarz E., Coons A.H. (1959). Specific Inhibition of Antibody Formation During Immunological Paralysis and Unresponsiveness. Nature.

[18] Zajac A.J., Blattman J.N., Murali-Krishna K., Sourdive D.J., Suresh M., Altman J.D., Ahmed R. (1998). Viral immune evasion due to persistence of activated T cells without effector function. J. Exp. Med..

[19] Wherry E.J., Blattman J.N., Murali-Krishna K., van der Most R., Ahmed R. (2003). Viral persistence alters CD8 T-cell immunodominance and tissue distribution and results in distinct stages of functional impairment. J. Virol..

[20] Doering T.A., Crawford A., Angelosanto J.M., Paley M.A., Ziegler C.G., Wherry E.J. (2012). Network analysis reveals centrally connected genes and pathways involved in CD8+ T cell exhaustion versus memory. Immunity.

[21] Khan O., Giles J.R., McDonald S., Manne S., Ngiow S.F., Patel K.P., Werner M.T., Huang A.C., Alexander K.A., Wu J.E. (2019). TOX transcriptionally and epigenetically programs CD8^+^ T cell exhaustion. Nature.

[22] Alfei F., Kanev K., Hofmann M., Wu M., Ghoneim H.E., Roelli P., Utzschneider D.T., von Hoesslin M., Cullen J.G., Fan Y. (2019). TOX reinforces the phenotype and longevity of exhausted T cells in chronic viral infection. Nature.

[23] Scott A.C., Dündar F., Zumbo P., Chandran S.S., Klebanoff C.A., Shakiba M., Trivedi P., Menocal L., Appleby H., Camara S. (2019). TOX is a critical regulator of tumour-specific T cell differentiation. Nature.

[24] Huang Y.J., Ngiow S.F., Baxter A.E., Manne S., Park S.L., Wu J.E., Khan O., Giles J.R., Wherry E.J. (2025). Continuous expression of TOX safeguards exhausted CD8 T cell epigenetic fate. Sci. Immunol..

[25] Seo H., Chen J., González-Avalos E., Samaniego-Castruita D., Das A., Wang Y.H., López-Moyado I.F., Georges R.O., Zhang W., Onodera A. (2019). TOX and TOX2 transcription factors cooperate with NR4A transcription factors to impose CD8^+^ T cell exhaustion. Proc. Natl. Acad. Sci. USA.

[26] Philip M., Fairchild L., Sun L., Horste E.L., Camara S., Shakiba M., Scott A.C., Viale A., Lauer P., Merghoub T. (2017). Chromatin states define tumour-specific T cell dysfunction and reprogramming. Nature.

[27] Rudloff M.W., Zumbo P., Favret N.R., Roetman J.J., Detrés Román C.R., Erwin M.M., Murray K.A., Jonnakuti S.T., Dündar F., Betel D. (2023). Hallmarks of CD8^+^ T cell dysfunction are established within hours of tumor antigen encounter before cell division. Nat. Immunol..

[28] Abdel-Hakeem M.S., Manne S., Beltra J.-C., Stelekati E., Chen Z., Nzingha K., Ali M.-A., Johnson J.L., Giles J.R., Mathew D. (2021). Epigenetic scarring of exhausted T cells hinders memory differentiation upon eliminating chronic antigenic stimulation. Nat. Immunol..

[29] Gurdon J.B., Laskey R.A., Reeves O.R. (1975). The developmental capacity of nuclei transplanted from keratinized skin cells of adult frogs. J. Embryol. Exp. Morphol..

[30] Wilmut I., Schnieke A.E., McWhir J., Kind A.J., Campbell K.H. (1997). Viable offspring derived from fetal and adult mammalian cells. Nature.

[31] Wakayama T., Perry A.C., Zuccotti M., Johnson K.R., Yanagimachi R. (1998). Full-term development of mice from enucleated oocytes injected with cumulus cell nuclei. Nature.

[32] Cowan C.A., Atienza J., Melton D.A., Eggan K. (2005). Nuclear reprogramming of somatic cells after fusion with human embryonic stem cells. Science.

[33] Yu J., Vodyanik M.A., He P., Slukvin I.I., Thomson J.A. (2006). Human embryonic stem cells reprogram myeloid precursors following cell-cell fusion. Stem Cells.

[34] Hochedlinger K., Jaenisch R. (2002). Monoclonal mice generated by nuclear transfer from mature B and T donor cells. Nature.

[35] Inoue K., Wakao H., Ogonuki N., Miki H., Seino K., Nambu-Wakao R., Noda S., Miyoshi H., Koseki H., Taniguchi M. (2005). Generation of cloned mice by direct nuclear transfer from natural killer T cells. Curr. Biol..

[36] Hochedlinger K., Jaenisch R. (2006). Nuclear reprogramming and pluripotency. Nature.

[37] Takahashi K., Yamanaka S. (2006). Induction of pluripotent stem cells from mouse embryonic and adult fibroblast cultures by defined factors. Cell.

[38] Takahashi K., Tanabe K., Ohnuki M., Narita M., Ichisaka T., Tomoda K., Yamanaka S. (2007). Induction of pluripotent stem cells from adult human fibroblasts by defined factors. Cell.

[39] Yu J., Vodyanik M.A., Smuga-Otto K., Antosiewicz-Bourget J., Frane J.L., Tian S., Nie J., Jonsdottir G.A., Ruotti V., Stewart R. (2007). Induced pluripotent stem cell lines derived from human somatic cells. Science.

[40] Maherali N., Sridharan R., Xie W., Utikal J., Eminli S., Arnold K., Stadtfeld M., Yachechko R., Tchieu J., Jaenisch R. (2007). Directly reprogrammed fibroblasts show global epigenetic remodeling and widespread tissue contribution. Cell Stem Cell.

[41] Park I.H., Zhao R., West J.A., Yabuuchi A., Huo H., Ince T.A., Lerou P.H., Lensch M.W., Daley G.Q. (2008). Reprogramming of human somatic cells to pluripotency with defined factors. Nature.

[42] Wernig M., Meissner A., Foreman R., Brambrink T., Ku M., Hochedlinger K., Bernstein B.E., Jaenisch R. (2007). In vitro reprogramming of fibroblasts into a pluripotent ES-cell-like state. Nature.

[43] Loh Y.H., Hartung O., Li H., Guo C., Sahalie J.M., Manos P.D., Urbach A., Heffner G.C., Grskovic M., Vigneault F. (2010). Reprogramming of T cells from human peripheral blood. Cell Stem Cell.

[44] Hong H., Takahashi K., Ichisaka T., Aoi T., Kanagawa O., Nakagawa M., Okita K., Yamanaka S. (2009). Suppression of induced pluripotent stem cell generation by the p53-p21 pathway. Nature.

[45] Nishimura K., Sano M., Ohtaka M., Furuta B., Umemura Y., Nakajima Y., Ikehara Y., Kobayashi T., Segawa H., Takayasu S. (2011). Development of defective and persistent Sendai virus vector: A unique gene delivery/expression system ideal for cell reprogramming. J. Biol. Chem..

[46] Seki T., Yuasa S., Oda M., Egashira T., Yae K., Kusumoto D., Nakata H., Tohyama S., Hashimoto H., Kodaira M. (2010). Generation of induced pluripotent stem cells from human terminally differentiated circulating T cells. Cell Stem Cell.

[47] Crompton J.G., Clever D., Vizcardo R., Rao M., Restifo N.P. (2014). Reprogramming antitumor immunity. Trends Immunol..

[48] Vizcardo R., Masuda K., Yamada D., Ikawa T., Shimizu K., Fujii S., Koseki H., Kawamoto H. (2013). Regeneration of human tumor antigen-specific T cells from iPSCs derived from mature CD8^+^ T cells. Cell Stem Cell.

[49] Saito H., Okita K., Fusaki N., Sabel M.S., Chang A.E., Ito F. (2016). Reprogramming of Melanoma Tumor-Infiltrating Lymphocytes to Induced Pluripotent Stem Cells. Stem Cells Int..

[50] Dudley M.E., Wunderlich J.R., Shelton T.E., Even J., Rosenberg S.A. (2003). Generation of tumor-infiltrating lymphocyte cultures for use in adoptive transfer therapy for melanoma patients. J. Immunother..

[51] Islam S.M.R., Maeda T., Tamaoki N., Good M.L., Kishton R.J., Paria B.C., Yu Z., Bosch-Marce M., Bedanova N.M., Liu C. (2023). Reprogramming of Tumor-reactive Tumor-infiltrating Lymphocytes to Human-induced Pluripotent Stem Cells. Cancer Res. Commun..

[52] Eminli S., Foudi A., Stadtfeld M., Maherali N., Ahfeldt T., Mostoslavsky G., Hock H., Hochedlinger K. (2009). Differentiation stage determines potential of hematopoietic cells for reprogramming into induced pluripotent stem cells. Nat. Genet..

[53] Churko J.M., Lee J., Ameen M., Gu M., Venkatasubramanian M., Diecke S., Sallam K., Im H., Wang G., Gold J.D. (2017). Transcriptomic and epigenomic differences in human induced pluripotent stem cells generated from six reprogramming methods. Nat. Biomed. Eng..

[54] Bulliard Y., Andersson B.S., Baysal M.A., Damiano J., Tsimberidou A.M. (2023). Reprogramming T cell differentiation and exhaustion in CAR-T cell therapy. J. Hematol. Oncol..

[55] Ji S., Xiong M., Chen H., Liu Y., Zhou L., Hong Y., Wang M., Wang C., Fu X., Sun X. (2023). Cellular rejuvenation: Molecular mechanisms and potential therapeutic interventions for diseases. Signal Transduct. Target. Ther..

[56] Nishimura T., Kaneko S., Kawana-Tachikawa A., Tajima Y., Goto H., Zhu D., Nakayama-Hosoya K., Iriguchi S., Uemura Y., Shimizu T. (2013). Generation of rejuvenated antigen-specific T cells by reprogramming to pluripotency and redifferentiation. Cell Stem Cell.

[57] Maeda T., Nagano S., Ichise H., Kataoka K., Yamada D., Ogawa S., Koseki H., Kitawaki T., Kadowaki N., Takaori-Kondo A. (2016). Regeneration of CD8αβ T Cells from T-cell-Derived iPSC Imparts Potent Tumor Antigen-Specific Cytotoxicity. Cancer Res..

[58] Galic Z., Kitchen S.G., Kacena A., Subramanian A., Burke B., Cortado R., Zack J.A. (2006). T lineage differentiation from human embryonic stem cells. Proc. Natl. Acad. Sci. USA.

[59] Deftos M.L., Bevan M.J. (2000). Notch signaling in T cell development. Curr. Opin. Immunol..

[60] Izon D.J., Punt J.A., Xu L., Karnell F.G., Allman D., Myung P.S., Boerth N.J., Pui J.C., Koretzky G.A., Pear W.S. (2001). Notch1 regulates maturation of CD4^+^ and CD8^+^ thymocytes by modulating TCR signal strength. Immunity.

[61] Schmitt T.M., Zúñiga-Pflücker J.C. (2002). Induction of T cell development from hematopoietic progenitor cells by delta-like-1 in vitro. Immunity.

[62] Timmermans F., Velghe I., Vanwalleghem L., De Smedt M., Van Coppernolle S., Taghon T., Moore H.D., Leclercq G., Langerak A.W., Kerre T. (2009). Generation of T cells from human embryonic stem cell-derived hematopoietic zones. J. Immunol..

[63] Chang C.W., Lai Y.S., Lamb L.S., Townes T.M. (2014). Broad T-cell receptor repertoire in T-lymphocytes derived from human induced pluripotent stem cells. PLoS ONE.

[64] Huijskens M.J., Walczak M., Koller N., Briedé J.J., Senden-Gijsbers B.L., Schnijderberg M.C., Bos G.M., Germeraad W.T. (2014). Technical advance: Ascorbic acid induces development of double-positive T cells from human hematopoietic stem cells in the absence of stromal cells. J. Leukoc. Biol..

[65] Kawai Y., Kawana-Tachikawa A., Kitayama S., Ueda T., Miki S., Watanabe A., Kaneko S. (2021). Generation of highly proliferative, rejuvenated cytotoxic T cell clones through pluripotency reprogramming for adoptive immunotherapy. Mol. Ther..

[66] Mohtashami M., Brauer P.M., Zúñiga-Pflücker J.C. (2023). Induction of Human T Cell Development In Vitro with OP9-DL4-7FS Cells Expressing Human Cytokines. Methods Mol. Biol..

[67] Iriguchi S., Yasui Y., Kawai Y., Arima S., Kunitomo M., Sato T., Ueda T., Minagawa A., Mishima Y., Yanagawa N. (2021). A clinically applicable and scalable method to regenerate T-cells from iPSCs for off-the-shelf T-cell immunotherapy. Nat. Commun..

[68] Minagawa A., Yoshikawa T., Yasukawa M., Hotta A., Kunitomo M., Iriguchi S., Takiguchi M., Kassai Y., Imai E., Yasui Y. (2018). Enhancing T Cell Receptor Stability in Rejuvenated iPSC-Derived T Cells Improves Their Use in Cancer Immunotherapy. Cell Stem Cell.

[69] van Oers N.S., Teh S.J., Garvin A.M., Forbush K.A., Perlmutter R.M., Teh H.S. (1993). CD8 inhibits signal transduction through the T cell receptor in CD4-CD8- thymocytes from T cell receptor transgenic mice reconstituted with a transgenic CD8 alpha molecule. J. Immunol..

[70] Nakayama K., Nakayama K., Negishi I., Kuida K., Louie M.C., Kanagawa O., Nakauchi H., Loh D.Y. (1994). Requirement for CD8 beta chain in positive selection of CD8-lineage T cells. Science.

[71] Vizcardo R., Klemen N.D., Islam S.M.R., Gurusamy D., Tamaoki N., Yamada D., Koseki H., Kidder B.L., Yu Z., Jia L. (2018). Generation of Tumor Antigen-Specific iPSC-Derived Thymic Emigrants Using a 3D Thymic Culture System. Cell Rep..

[72] Seet C.S., He C., Bethune M.T., Li S., Chick B., Gschweng E.H., Zhu Y., Kim K., Kohn D.B., Baltimore D. (2017). Generation of mature T cells from human hematopoietic stem and progenitor cells in artificial thymic organoids. Nat. Methods.

[73] Montel-Hagen A., Seet C.S., Li S., Chick B., Zhu Y., Chang P., Tsai S., Sun V., Lopez S., Chen H.C. (2019). Organoid-Induced Differentiation of Conventional T Cells from Human Pluripotent Stem Cells. Cell Stem Cell.

[74] Montel-Hagen A., Sun V., Casero D., Tsai S., Zampieri A., Jackson N., Li S., Lopez S., Zhu Y., Chick B. (2020). In Vitro Recapitulation of Murine Thymopoiesis from Single Hematopoietic Stem Cells. Cell Rep..

[75] Jing R., Scarfo I., Najia M.A., Lummertz da Rocha E., Han A., Sanborn M., Bingham T., Kubaczka C., Jha D.K., Falchetti M. (2022). EZH1 repression generates mature iPSC-derived CAR T cells with enhanced antitumor activity. Cell Stem Cell.

[76] Jing R., Falchetti M., Han T., Najia M., Hensch L.T., Meader E., Lummertz da Rocha E., Kononov M., Wang S., Bingham T. (2025). Maturation and persistence of CAR T cells derived from human pluripotent stem cells via chemical inhibition of G9a/GLP. Cell Stem Cell.

[77] Themeli M., Kloss C.C., Ciriello G., Fedorov V.D., Perna F., Gonen M., Sadelain M. (2013). Generation of tumor-targeted human T lymphocytes from induced pluripotent stem cells for cancer therapy. Nat. Biotechnol..

[78] Tran E., Robbins P.F., Lu Y.C., Prickett T.D., Gartner J.J., Jia L., Pasetto A., Zheng Z., Ray S., Groh E.M. (2016). T-Cell Transfer Therapy Targeting Mutant KRAS in Cancer. N. Engl. J. Med..

[79] Leidner R., Sanjuan Silva N., Huang H., Sprott D., Zheng C., Shih Y.P., Leung A., Payne R., Sutcliffe K., Cramer J. (2022). Neoantigen T-Cell Receptor Gene Therapy in Pancreatic Cancer. N. Engl. J. Med..

[80] Leko V., Rosenberg S.A. (2020). Identifying and Targeting Human Tumor Antigens for T Cell-Based Immunotherapy of Solid Tumors. Cancer Cell.

[81] Peri A., Salomon N., Wolf Y., Kreiter S., Diken M., Samuels Y. (2023). The landscape of T cell antigens for cancer immunotherapy. Nat. Cancer.

[82] de Rham C., Villard J. (2014). Potential and limitation of HLA-based banking of human pluripotent stem cells for cell therapy. J. Immunol. Res..

[83] Kitayama S., Zhang R., Liu T.Y., Ueda N., Iriguchi S., Yasui Y., Kawai Y., Tatsumi M., Hirai N., Mizoro Y. (2016). Cellular Adjuvant Properties, Direct Cytotoxicity of Re-differentiated Vα24 Invariant NKT-like Cells from Human Induced Pluripotent Stem Cells. Stem Cell Rep..

[84] Zeng J., Tang S.Y., Wang S. (2019). Derivation of mimetic γδ T cells endowed with cancer recognition receptors from reprogrammed γδ T cell. PLoS ONE.

[85] Maeda T., Nagano S., Kashima S., Terada K., Agata Y., Ichise H., Ohtaka M., Nakanishi M., Fujiki F., Sugiyama H. (2020). Regeneration of Tumor-Antigen-Specific Cytotoxic T Lymphocytes from iPSCs Transduced with Exogenous TCR Genes. Mol. Ther. Methods Clin. Dev..

[86] Kim K., Doi A., Wen B., Ng K., Zhao R., Cahan P., Kim J., Aryee M.J., Ji H., Ehrlich L.I. (2010). Epigenetic memory in induced pluripotent stem cells. Nature.

[87] Nagano S., Maeda T., Ichise H., Kashima S., Ohtaka M., Nakanishi M., Kitawaki T., Kadowaki N., Takaori-Kondo A., Masuda K. (2020). High Frequency Production of T Cell-Derived iPSC Clones Capable of Generating Potent Cytotoxic T Cells. Mol. Ther. Methods Clin. Dev..

[88] Niizuma K., Nishimura T., Villanueva J., Amaya L., Fowler J.L., Isobe T., Nakauchi Y., Saavedra B., Xu H., Nakanishi M. (2025). Development of iPSC-Derived T Cells Targeting EGFR Neoantigens in Non-Small Cell Lung Cancer. Mol. Ther. Methods Clin. Dev..

[89] Kashima S., Maeda T., Masuda K., Nagano S., Inoue T., Takeda M., Kono Y., Kobayashi T., Saito S., Higuchi T. (2020). Cytotoxic T Lymphocytes Regenerated from iPS Cells Have Therapeutic Efficacy in a Patient-Derived Xenograft Solid Tumor Model. iScience.

[90] Chang P.C., Yuan X., Zampieri A., Towns C., Yoo S.P., Engstrom C., Tsai S., Robles C.R., Zhu Y., Lopez S. (2024). Generation of antigen-specific mature T cells from RAG1^−/−^RAG2^−/−^B2M^−/−^ stem cells by engineering their microenvironment. Nat. Biomed. Eng..

[91] Minguet S., Maus M.V., Schamel W.W. (2025). From TCR fundamental research to innovative chimeric antigen receptor design. Nat. Rev. Immunol..

[92] Turtle C.J., Hanafi L.A., Berger C., Gooley T.A., Cherian S., Hudecek M., Sommermeyer D., Melville K., Pender B., Budiarto T.M. (2016). CD19 CAR-T cells of defined CD4^+^:CD8^+^ composition in adult B cell ALL patients. J. Clin. Investig..

[93] Sommermeyer D., Hudecek M., Kosasih P.L., Gogishvili T., Maloney D.G., Turtle C.J., Riddell S.R. (2016). Chimeric antigen receptor-modified T cells derived from defined CD8^+^ and CD4^+^ subsets confer superior antitumor reactivity in vivo. Leukemia.

[94] Melenhorst J.J., Chen G.M., Wang M., Porter D.L., Chen C., Collins M.A., Gao P., Bandyopadhyay S., Sun H., Zhao Z. (2022). Decade-long leukaemia remissions with persistence of CD4^+^ CAR T cells. Nature.

[95] Ohoka Y., Kuwata T., Tozawa Y., Zhao Y., Mukai M., Motegi Y., Suzuki R., Yokoyama M., Iwata M. (1996). In vitro differentiation and commitment of CD4^+^ CD8^+^ thymocytes to the CD4 lineage, without TCR engagement. Int. Immunol..

[96] Fong H., Mendel M., Jascur J., Najmi L., Kim K., Lew G., Garimalla S., Schock S., Hu J., Villegas A.G. (2025). A Serum- and Feeder-Free System to Generate CD4 and Regulatory T Cells from Human iPSCs. Stem Cells.

[97] Wildt K.F., Sun G., Grueter B., Fischer M., Zamisch M., Ehlers M., Bosselut R. (2007). The transcription factor Zbtb7b promotes CD4 expression by antagonizing Runx-mediated activation of the CD4 silencer. J. Immunol..

[98] Ishiguro Y., Iriguchi S., Asano S., Shinohara T., Shiina S., Arima S., Kassai Y., Sakai Y., Obama K., Kaneko S. (2023). Lineage tracing of T cell differentiation from T-iPSC by 2D feeder-free culture and 3D organoid culture. Front. Immunol..

[99] Hoang D.M., Pham P.T., Bach T.Q., Ngo A.T.L., Nguyen Q.T., Phan T.T.K., Nguyen G.H., Le P.T.T., Hoang V.T., Forsyth N.R. (2022). Stem cell-based therapy for human diseases. Signal Transduct. Target. Ther..

[100] Hosking M.P., Shirinbak S., Omilusik K., Chandra S., Kaneko M.K., Gentile A., Yamamoto S., Shrestha B., Grant J., Boyett M. (2025). Preferential tumor targeting of HER2 by iPSC-derived CAR T cells engineered to overcome multiple barriers to solid tumor efficacy. Cell Stem Cell.

[101] Baulu E., Gardet C., Chuvin N., Depil S. (2023). TCR-engineered T cell therapy in solid tumors: State of the art and perspectives. Sci. Adv..

[102] Kirkeby A., Main H., Carpenter M. (2025). Pluripotent stem-cell-derived therapies in clinical trial: A 2025 update. Cell Stem Cell.

[103] Moy A.B., Kamath A., Ternes S., Kamath J. (2023). The Challenges to Advancing Induced Pluripotent Stem Cell-Dependent Cell Replacement Therapy. Med. Res. Arch..

[104] Llorente I.L., Hatanaka E.A., Meadow M.E., Xie Y., Lowry W.E., Carmichael S.T. (2021). Reliable generation of glial enriched progenitors from human fibroblast-derived iPSCs. Stem Cell Res..

[105] Warren L., Manos P.D., Ahfeldt T., Loh Y.H., Li H., Lau F., Ebina W., Mandal P.K., Smith Z.D., Meissner A. (2010). Highly efficient reprogramming to pluripotency and directed differentiation of human cells with synthetic modified mRNA. Cell Stem Cell.

[106] Rubio-Casillas A., Cowley D., Raszek M., Uversky V.N., Redwan E.M. (2024). Review: N1-methyl-pseudouridine (m1Ψ): Friend or foe of cancer?. Int. J. Biol. Macromol..

[107] Mulroney T.E., Pöyry T., Yam-Puc J.C., Rust M., Harvey R.F., Kalmar L., Horner E., Booth L., Ferreira A.P., Stoneley M. (2024). N^1^-methylpseudouridylation of mRNA causes +1 ribosomal frameshifting. Nature.

[108] Morais P., Adachi H., Yu Y.T. (2021). The Critical Contribution of Pseudouridine to mRNA COVID-19 Vaccines. Front. Cell Dev. Biol..

[109] Pepini T., Pulichino A.M., Carsillo T., Carlson A.L., Sari-Sarraf F., Ramsauer K., Debasitis J.C., Maruggi G., Otten G.R., Geall A.J. (2017). Induction of an IFN-Mediated Antiviral Response by a Self-Amplifying RNA Vaccine: Implications for Vaccine Design. J. Immunol..

[110] Wang J., Sun S., Deng H. (2023). Chemical reprogramming for cell fate manipulation: Methods, applications, and perspectives. Cell Stem Cell.

[111] Ando M., Nishimura T., Yamazaki S., Yamaguchi T., Kawana-Tachikawa A., Hayama T., Nakauchi Y., Ando J., Ota Y., Takahashi S. (2015). A Safeguard System for Induced Pluripotent Stem Cell-Derived Rejuvenated T Cell Therapy. Stem Cell Rep..

